# Long-term GLP-1 receptor agonist therapy in obesity: expert panel recommendations for good clinical practice

**DOI:** 10.3389/fendo.2026.1907270

**Published:** 2026-09-28

**Authors:** Davide Brancato, Giorgio Borretta, Marco Chianelli, Olga Disoteo, Marco Grasso, Franco Grimaldi, Edoardo Guastamacchia, Fabrizio Muratori, Salvatore Oleandri, Marcello Orio, Paola Pantanetti, Enrico Papini, Agnese Persichetti, Roberto Vettor, Federica Vinciguerra

**Affiliations:** 1 Department of Internal Medicine, Casa di Cura Candela, Palermo, Italy; 2 Department of Endocrinology, Diabetes and Metabolism, Santa Croce & Carle Hospital, Cuneo, Italy; 3 Unit of Endocrinology and Metabolism, Regina Apostolorum Hospital, Albano, Rome, Italy; 4 SC Diabetologia, ASST Grande Ospedale Metropolitano Niguarda, Milan, Italy; 5 Unità Operativa Complessa (UOC) Malattie Endocrine, del Ricambio e della Nutrizione, AULSS 2 Marca Trevigiana, Treviso, Italy; 6 Endocrinology Unit, Diseases of Metabolism and Clinical Nutrition Unit, University Hospital S.M. Misericordia, Udine, Italy; 7 Section of Internal Medicine, Geriatrics, Endocrinology and Rare Diseases, Interdisciplinary Department of Medicine, University of Bari “Aldo Moro”, Bari, Italy; 8 Division of Endocrinology, Diabetology and Clinical Nutrition, Sant’Anna Hospital - ASST Lariana, Como, Italy; 9 Endocrinology and Metabolism Unit, Azienda Sanitaria Locale (ASL) CITTA‘ di Torino, Turin, Italy; 10 Medical and Diabetological Center Centro Medico Specialistico Orio (CMSO), Salerno, Italy; 11 Azienda Sanitaria Territoriale (AST) Fermo, Unit of Diabetology, Fermo, Italy; 12 Endocrinology and Metabolism, Regina Apostolorum Hospital., Roma, Italy; 13 Ministry of Interior - Department of Firefighters, Public Rescue and Civil Defense, Rome, Italy; 14 Internal Medicine, Center for the Study and the Integrated Treatment of Obesity, Department of Medicine, University of Padova, Padua, Italy; 15 Endocrinology Section, Department of Clinical and Experimental Medicine, University of Catania, Catania, Italy

**Keywords:** cardiometabolic benefits, GLP-1 receptor agonists, long-term therapy, obesity, real-world management, weight maintenance

## Abstract

Glucagon-like peptide-1 receptor agonists (GLP-1RAs) have emerged as highly effective therapies for obesity, facilitating weight loss and improving cardiometabolic risk factors. However, several challenges persist regarding optimal long-term management in real-world clinical practice. A board of clinicians with expertise in obesity and metabolic diseases discussed practical recommendations for the sustainable long-term management of GLP-1RA therapy. Key themes included the maintenance of clinical benefits, prevention of weight regain, treatment sustainability, and long-term safety and tolerability. The panel highlighted the necessity of approaching obesity as a chronic, relapsing disease requiring long-term therapeutic strategies. Comprehensive baseline phenotyping and a treat-to-benefit approach were identified as essential for defining clinically meaningful treatment targets beyond weight loss alone. Maintenance therapy after achievement of initial outcomes, flexible dose management, and early recognition of relapse signals were considered essential for preserving benefits and preventing weight regain. Pharmacotherapy should be integrated within a multimodal care model that includes nutritional counseling, physical activity, and behavioral support, while addressing real-world barriers such as economic sustainability. Proactive management of adverse events and patient education were recognized as critical for ensuring long-term adherence. Sustainable use of GLP-1RA therapy requires integrated, patient-centered management strategies that combine pharmacological treatment with lifestyle interventions and pragmatic dose adjustment. Future collaborative real-world data initiatives may further refine these practical recommendations and help optimize long-term obesity care.

## Introduction

Obesity is a chronic, relapsing disease associated with a substantial burden of cardiometabolic complications and reduced life expectancy (1–3). Over the past decade, glucagon-like peptide-1 receptor agonists (GLP-1RAs) have emerged as highly effective pharmacological tools for the management of obesity, demonstrating robust weight reduction together with improvements in several cardiometabolic parameters (4, 5). However, the growing availability and use of these agents have also highlighted several unresolved challenges, particularly regarding long-term treatment strategies, sustainability, and maintenance of clinical benefits (6–8).

While randomized clinical trials provide strong evidence of long-term efficacy and safety (9, 10), the optimal real-world management of GLP-1RA therapy in obesity - especially beyond the initial weight-loss phase - remains a subject of ongoing clinical debate (6–8). Issues such as dose optimization, prevention of weight regain, long-term adherence, economic sustainability, and management of adverse events require pragmatic solutions that integrate scientific evidence with clinical experience (11–16).

The objective of this expert opinion article is to summarize the key insights that emerged from the discussion and translate them into practical clinical suggestions. Of note, these practical recommendations are intended to support clinical decision-making and do not replace locally approved prescribing information; treatment should be individualized based on patient characteristics, local availability, and reimbursement.

## Methods

To address these aspects, an advisory board composed by 15 clinicians with expertise in obesity and metabolic diseases convened to discuss current challenges and future directions in the long-term management of GLP-1RA therapy, drawing on their clinical experience. Dr. Davide Brancato selected a team of national experts based on their academic backgrounds and professional experience. The board included specialists in endocrinology or endocrinology-related disciplines, with additional expertise in diabetes and diabetology, metabolic diseases, clinical nutrition, osteoporosis, internal medicine, and nuclear medicine.

The work was conducted through a series of three virtual meetings, during which the panel engaged structured discussions focused on key domains of long-term GLP-1RA use. The discussions consistently addressed three main thematic areas: (i) the importance of weight maintenance and strategies to prevent weight regain; (ii) care models and therapeutic approaches to enhance the sustainability of long-term GLP-1RA therapy; and (iii) strategies for optimizing safety and tolerability over prolonged treatment durations. These topics were revisited and further refined across the three meetings to allow progressive development and alignment of expert perspectives.

Prior to each meeting, key topics were identified and shared with participants to facilitate focused discussion. During the sessions, experts contributed real-world clinical insights, discussed practical challenges, and explored potential solutions. The discussions were moderated to ensure balanced participation, comprehensive coverage of each topic and inclusion of any range of clinical perspectives. Indeed, while no formal voting process or quantitative assessment of agreement was performed, convergence of expert opinion was achieved through discussion and successive rounds of feedback. When divergent perspectives emerged, these were discussed during the meetings and the final suggestions were formulated to reflect the whole orientations represented in the panel.

Following the meetings, key messages and practical considerations emerging from the discussions were synthesized and progressively refined by the panel. The draft opinions were reviewed collectively to ensure that they accurately reflected the range of expert perspectives and clinical practices discussed during the meetings.

The resulting suggestions from the board are intended to support good clinical practice, with current practical knowledge in the long-term management of patients receiving GLP-1RA therapy.

The project did not receive any financial support beyond that which was declared in the funding disclosure. The unconditional sponsor played no part in the organization of the board, did not participate in meetings, and had no involvement in the development of the manuscript.

## Benefits maintenance and prevention of weight regain

Aiming to ensure the sustained maintenance of the benefits associated with GLP-1RAs in obesity management, the panel identified comprehensive baseline patient phenotyping as a critical first step. This assessment should extend beyond simple anthropometric measures - such as body mass index (BMI), body weight, waist circumference (WC), waist to height ratio (WHtR) - and include a broader evaluation of disease severity using validated tools such as the *Edmonton Obesity Staging System* (17). The panel also emphasized the importance of incorporating body composition assessment into routine clinical evaluation. While advanced imaging techniques such as dual-energy X-ray absorptiometry (DEXA) provide detailed information, their cost and logistical requirements often render them impractical for everyday clinical practice. Bioelectrical impedance analysis (BIA) is therefore considered a practical and accessible tool for monitoring changes in fat mass (FM), skeletal muscle mass (SMM) and hydration status throughout treatment (18).

In addition to physical metrics, baseline phenotyping must account for behavioral and psychological drivers of weight gain. Identifying eating patterns (e.g., emotional eating, binge eating, or night eating syndrome) at the outset allows for a more tailored pharmacological and behavioral approach. This holistic characterization at baseline not only assists in setting realistic patient expectations but also provides a robust clinical “anchor” to justify the continuation of treatment when weight loss reaches a plateau but metabolic benefits persist.

Understanding the reasons driving patients to seek treatment emerged as a fundamental component of the initial assessment (19). Individuals often present following a cardiovascular event or in the context of conditions such as polycystic ovary syndrome (PCOS), infertility, prediabetes, or other obesity correlated complications (ORCD) (19). By identifying these motivations, clinicians can define clinically meaningful therapeutic targets that extend beyond weight reduction alone. Depending on the clinical scenario, outcomes used to define treatment success may include:

- Systemic Inflammation: reduction in inflammatory biomarkers, such as high-sensitivity C-reactive protein (hs-CRP), which serves as a proxy for cardiovascular risk mitigation.
- Reproductive Health: restoration of menstrual regularity and improvement of ovulatory function in patients with PCOS.
- Glycemic Status: achieving enhanced glycemic control—or even the reversion to normoglycemia—in individuals with prediabetes.
- Hepatometabolic Health (**Appendix 2 in Supplementary Material**: Meaningful improvements in biochemical markers (e.g., liver enzymes) and instrumental parameters (e.g., elastography or imaging-based fat fraction) in patients with Metabolic Dysfunction-Associated Steatotic Liver Disease (MASLD) (20).

By aligning therapeutic goals with patient-specific comorbidities, clinicians shifts from prescribing generic weight-loss (“weight-centered” approach) to a personalized “treat-to-benefit” strategy. Indeed, evidence indicate that cardiometabolic and inflammatory parameters may continue to improve even after weight stabilization. Therefore, weight loss is considered primarily as an intermediate outcome rather than the sole indicator of therapeutic success. Conversely, many of these clinical benefits may rapidly diminish following treatment discontinuation (8, 12, 13, 15, 21).

Regardless of the individualized therapeutic targets defined for each patient, obesity should be managed according to its nature as a chronic, relapsing disease (22, 23). The treatment should be conceived as a long-term strategy rather than a short-term or cyclical intervention and this principle should be clearly communicated to patients from the outset (23, 24). Clinical experience and available evidence indicate that discontinuation of GLP-1RA therapy is frequently followed by meaningful weight regain, involving a substantial proportion of the previously lost weight within 12 months (13, 15, 21). This regain is associated with a partial return to baseline levels of several cardiometabolic parameters (8, 12). Repeated cycles of discontinuation and re-initiation may complicate long-term management; however, clinical evidence on this topic is limited and further research is needed to clarify the possible existence of a metabolic legacy–like effect.

Furthermore, the advisory board expressed a particularly strong position regarding patients with established cardiovascular disease, in whom any decision to discontinue should be made cautiously and individualized, considering cardiovascular risk and the evidence base for ongoing GLP-1RA therapy (4, 5, 25). In this context, established atherosclerotic cardiovascular disease refers to: prior myocardial infarction or coronary revascularization; coronary artery disease documented by angiography or coronary TC, or myocardial ischemia documented by non-invasive stress testing or vasospastic angina or microvascular angina; prior ischemic or hemorrhagic stroke or transitory ischemic attack or carotid artery stenosis > 50%; atherosclerotic aortic aneurysm; intermittent claudication with ankle-brachial index (ABI) <0.85 or previous peripheral arterial revascularization or amputation due to atherosclerotic disease. In these specific conditions, the potential cardiovascular benefits of continued treatment should be carefully weighed against factors that may warrant discontinuation, including clinically relevant or intolerable adverse effects, within an individualized assessment of the overall benefit–risk profile.

Maintenance therapy should therefore be considered an integral component of obesity management (6, 8, 13, 23, 26). Based on their experience, the expert panel suggest that once predefined therapeutic outcomes have been achieved at the maximum effective and tolerated dose, treatment should generally be continued to consolidate these benefits. Given the lack of evidence establishing the optimal treatment duration, the authors pragmatically suggest a maintenance period of approximately 6–12 months before considering dose reduction. When appropriate, dose de-escalation should occur gradually and flexibly (27, 28) and should be guided by a multidimensional assessment of clinical targets rather than by body weight alone (20). In this context, the availability of intermediate dose steps (e.g., for semaglutide) may facilitate individualized and flexible dose adjustment (29). Importantly, the re-emergence of hunger and the loss of satiety may represent early signals of impending weight regain and should prompt timely reassessment of treatment, including consideration of re-escalation to the previously effective dose.

Overall, obesity pharmacotherapy often requires long-term treatment, with periodic reassessment of treatment efficacy, tolerability, and the appropriateness of the maintenance dose, in accordance with locally approved prescribing information. From a pragmatic perspective, the panel considered that, in selected patients, individualized dose adjustment may be evaluated once body weight stability and clinically meaningful outcomes have been achieved with the aim of preserving clinically meaningful outcomes for as long as possible (“the longer the better”). The panel suggested a period of approximately six to twelve months of stable clinical response at the maximum tolerated dose before considering such an individualized approach. However, this timeframe and the concept of maintaining the lowest effective dose represent expert-derived pragmatic suggestions rather than evidence-based or universally approved dosing strategies; at this time, there is a lack of clinical evidence to determine the ideal treatment duration. Any dose reduction, reduced-frequency administration, or modification of dosing intervals outside locally approved prescribing information should therefore not be considered standard clinical practice.

Finally, the board recommends scheduling clinical and nutritional follow-up assessments, together with anthropometric and body composition measurements and laboratory evaluations (**Supplementary Table S1, Supplementary Material**). Practical suggestions for long-term maintenance of GLP-1RA therapy benefits are summarized in **Table 1**.

**Table 1:** Practical recommendations for sustainable long-term benefits, management, safety and tolerability of GLP-1RA therapy.

Long-term maintenance of GLP-1RA therapy benefits		
Clinical principle	Practical recommendation	Clinical rationale
Start with comprehensive phenotyping	Perform baseline phenotyping and define a limited set of measurable targets aligned with the patient’s clinical profile and treatment motivation (e.g., cardiometabolic, inflammatory, reproductive, or hepatic outcomes).	Treatment success should be defined by clinically meaningful benefits rather than weight loss alone.
Frame treatment as long-term therapy	Clearly communicate that obesity is a chronic, relapsing disease and that GLP-1RA therapy is generally intended for sustained management. Avoid unnecessary treatment interruption, particularly in patients with established cardiovascular disease.	Discontinuation is frequently associated with weight regain and reversal of cardiometabolic benefits.
Include a structured maintenance phase	After achieving therapeutic outcomes at the maximum tolerated effective dose, suggested maintenance period of about 6–12 months before considering dose reduction (currently, there is no clinical evidence to determine the optimal treatment duration).	Consolidating metabolic adaptations might potentially contribute to more stable outcomes and a lower risk of early relapse, although this remains to be established.
Use gradual, target-guided dose adjustment	When appropriate, reduce the dose progressively based on multidimensional clinical outcomes rather than body weight alone. Monitor early relapse signals such as return of hunger or loss of satiety.	Early detection of relapse allows timely dose re-escalation and helps preserve treatment benefits.
Consider individualized maintenance dose	Where consistent with the locally approved prescribing information, treatment may be maintained at the lowest approved dose that provides adequate clinical benefit.	Maintaining the minimum effective dose might potentially contribute to consolidating metabolic adaptations achieved during the weight-loss phase while balancing efficacy, tolerability, and long-term treatment sustainability, although this remains to be established.

Sustainable long-term management of GLP-1RA therapy		
Clinical principle	Practical recommendation	Clinical rationale
Establish a shared therapeutic agreement	At treatment initiation, define expectations, patient responsibilities, and criteria for treatment continuation or adjustment through clear communication and active patient involvement.	Shared decision-making improves patient engagement, strengthens adherence, and supports long-term treatment sustainability.
Personalize therapy within real-world constraints	Tailor treatment strategies by considering tolerability, lifestyle factors, and economic sustainability alongside clinical targets.	Real-world barriers frequently influence adherence; individualized strategies may help maintain treatment continuity over time.
Prioritize treatment continuity	When financial or tolerability constraints arise, consider individual strategies of dose reduction or adjustment of dosing intervals rather than complete treatment discontinuation. These strategies are not intended for standard clinical practice, but as a pragmatic option that could support and preserve the achieved clinical outcomes in selected cases.	Maintaining some degree of pharmacological coverage may preserve metabolic benefits and reduce the risk of rapid weight regain.
Maintain lifestyle support throughout treatment	Continue structured dietary counseling, promotion of physical activity, and behavioral interventions even during dose adjusting phases or in case of temporary pharmacological interruption.	Lifestyle interventions reinforce metabolic improvements and support long-term weight stability beyond pharmacological effects.

Long-term safety and tolerability of GLP-1RA therapy		
Clinical Principle	Practical Recommendation	Clinical Rationale
Educate patients from the start of therapy	Inform patients about expected side effects, their generally transient nature, and practical strategies to mitigate them. Individualized dosing strategies could be considered a valid component of treatment optimization.	Early education improves patient reassurance, supports adherence, and reduces unnecessary treatment discontinuation due to manageable adverse events.
Use individualized dosing strategies to manage gastrointestinal symptoms	When nausea or other gastrointestinal symptoms occur, consider individualized dosing strategies such as dose reduction, temporary titration pauses, or dose fractionation, to improve tolerability while maintaining treatment continuity.	Dose flexibility might potentially contribute to managing adverse events without compromising overall therapeutic benefit, although this remains to be established.
Do not consider nausea a therapeutic goal	If adverse effects persist or interfere with daily functioning, reassess therapy and adjust dosing accordingly.	The therapeutic objective of GLP-1RA therapy is satiety and metabolic benefit, not treatment-related discomfort.
Integrate nutritional counseling into adverse-event management	Advise patients to avoid large meals, limit fluid intake during meals, and identify dietary triggers that may worsen gastrointestinal symptoms.	Dietary adjustments can significantly improve tolerability and help reduce gastrointestinal side effects.
Support body composition during weight loss	Combine GLP-1RA therapy with appropriate assessment of nutritional status and individualized, risk-based evaluation of bone health through biochemical and instrumental examinations (example: BIA, DEXA) when clinically indicated, alongside nutritional guidance and resistance-based physical activity.	Supporting the preservation of SMM and bone health while reducing FM helps optimize body composition and metabolic health during weight loss.

## Sustainable long-term management models for GLP-1RA therapy

Ensuring the long-term sustainability of GLP-1RA therapy requires an integrated and pragmatic management model that balances clinical efficacy, patient adherence, and economic feasibility. In line with current guidelines and the available literature, the advisory board emphasized that pharmacological treatment should not be considered in isolation but rather as part of a comprehensive care pathway that includes structured nutritional counseling, promotion of physical activity, and continuous patient education (23, 30–33).

Within this integrated framework, the board stressed the importance of establishing a clear therapeutic agreement with the patient at the beginning of treatment. This shared framework should define expectations, responsibilities, and the conditions under which therapy may be continued, adjusted, or intensified. In this light, also economic issues - such as long-term costs, reimbursement status, medication availability, and the patient’s ability to sustain therapy over time sustainability - should be discussed at treatment initiation. This point should be addressed proactively as part of the therapeutic plan rather than only after financial difficulties emerge. Treatment plans should also account for the indirect costs of care, including travel, repeated visits, laboratory testing, and body composition assessments. A periodic reassessment of these considerations is suggested, as changes in employment, insurance coverage, family circumstances, or healthcare policies may affect treatment continuity.

Based on the experience of the involved specialists, the panel underscored the need to move beyond the rigid conditions of clinical trials and adopt a pragmatic approach to therapy personalization that reflects real-world constraints, including tolerability, lifestyle factors, and economic sustainability (6, 7, 24, 34). The sustainability of therapy may vary substantially across healthcare settings: patients with type 2 diabetes treated within public healthcare systems often benefit from reimbursement policies that facilitate long-term access to therapy, whereas individuals treated primarily for obesity in private settings frequently face greater economic barriers. In Italy, this disparity has stimulated ongoing discussion among policymakers and clinical stakeholders regarding potential reimbursement pathways for obesity pharmacotherapy, particularly for patients with significant comorbidities or elevated cardiometabolic risk.

While awaiting broader reimbursement strategies, the advisory board agreed that maintaining some degree of pharmacological coverage should be prioritized whenever possible.

When financial or tolerability constraints arise, dose reduction may represent a pragmatic alternative to avoid complete treatment discontinuation (35). Even at reduced doses, GLP-1RA therapy may continue to exert metabolically protective effects and help preserve part of the clinical benefits previously achieved (27, 28). Real-world observations suggest that, in some patients, clinically meaningful and sustained outcomes may be obtained at intermediate approved doses; however, patients should be informed that clinical evidence supporting specific cost-driven de-escalation strategies remains limited and that close clinical monitoring is required (28). Follow-up should be organized according to clinical risk and treatment phase. Stable patients may not require the same intensity of monitoring as patients undergoing titration, experiencing adverse events, or showing early signs of weight regain. Clinical visits, laboratory evaluations, and body composition measurements should therefore be prioritized according to their expected impact on management, while preserving essential safety monitoring.

Treatment targets should be considered dynamic and adaptable over time, reflecting changes in clinical status, comorbidities, and patient priorities. Active patient involvement was therefore regarded as essential, and effective communication itself was recognized as a key therapeutic that reinforce adherence and long-term engagement. The authors agree that continuity of care should be maintained even when medication doses are reduced or temporarily suspended. Structured dietary support, regular physical activity, and behavioral interventions remain essential for long-term obesity management (23, 30–33). Cognitive-behavioral strategies and self-monitoring techniques are considered a valuable support for adherence, especially during phases in which pharmacological efficacy may be attenuated. The panel further noted that GLP-1RA therapy may function as a form of “metabolic educator,” helping patients develop greater awareness of the physiological consequences of dietary excess and reinforcing healthier eating behaviors. In selected cases - particularly among younger individuals with mild obesity who maintain high levels of physical activity - treatment discontinuation after sustained maintenance of lifestyle changes may be possible without significant regaining weight. However, this represents an expert-derived clinical observation and has not been prospectively validated; currently, no established clinical phenotype, including these characteristics, can reliably predict successful treatment withdrawal. The panel therefore pointed out that – in their clinical experience – such situations should be regarded as exceptions rather than the general rule in obesity management.

Practical suggestions for sustainable long-term management of GLP-1RA therapy are summarized in **Table 1**.

Looking ahead, the advisory board noted that the evolving availability and regulatory approval of oral GLP-1RAs for obesity treatment (36–38) may further improve the long-term sustainability and acceptability of pharmacological treatment. In the panel’s view, oral formulations could help reduce some of the practical barriers currently associated with injectable therapies, potentially providing additional options for long-term treatment, where approved and clinically appropriate. Beyond improving treatment acceptability, oral GLP-1 receptor agonists may offer new opportunities for long-term maintenance strategies. The panel hypothesized that, once clinically meaningful weight loss and metabolic targets have been achieved with injectable therapy, transitioning selected patients to an approved oral formulation might represent a potential maintenance approach; however, this strategy has not been prospectively validated and should not currently be considered an established step-down strategy. Following achievement of clinically meaningful weight loss with injectable therapies, transitioning to oral GLP-1 receptor agonists warrants investigation as a potential long-term strategy to preserve metabolic benefits while minimizing treatment burden, potentially improving long term adherence and economic sustainability. Whether such a transition could safely facilitate dose de-escalation or reduced pharmacological exposure also remains to be established. Rather than representing simply an alternative route of administration, oral GLP-1 receptor agonists could potentially expand future options for the maintenance phase of obesity treatment. Prospective studies are needed to establish the efficacy, safety, appropriate patient selection, and optimal implementation of injectable-to-oral transition strategies. Therefore, this approach should currently be regarded as a hypothesis for future investigation rather than an established management strategy.

## Long-term safety and tolerability

Long-term safety and tolerability represent fundamental prerequisites for the chronic use of GLP-1RAs in obesity management (39–41). The advisory board agreed that pharmacological treatment should enhance patients’ quality of life and that proactive prevention and management of adverse events are essential to sustain long-term adherence.

Patient education should begin at the first clinical encounter. Individuals should be informed about the potential adverse effects of therapy, reassured about their generally transient nature, and instructed on practical strategies to mitigate them (42–45). Importantly, patients should understand that dose adjustments represent a legitimate and effective tool for managing tolerability and should not be interpreted as treatment failure (43, 45, 46).

In line with the literature and prescribing information, dose flexibility was indeed identified as one of the most effective strategies for managing gastrointestinal adverse events, particularly nausea (43, 45, 46). The clinical experience of the board suggests that slower titration, temporary titration pauses, or temporary dose reduction may help manage gastrointestinal adverse events and support treatment continuation.

A key message by the advisory board was that nausea should never be considered a desirable or necessary effect of GLP-1RA therapy. The therapeutic objective is the induction of satiety, not discomfort. Persistent adverse effects that interfere with daily functioning are unacceptable in the context of long-term therapy and should always prompt clinical reassessment and appropriate dose adjustment. At the same time, the board acknowledged that mild nausea may occur during the early phases of treatment with GLP-1RAs and typically subsides with continued therapy (47–49). Interestingly, the reappearance of nausea after a period of stability was indicated as a potential clinical signal of suboptimal adherence, particularly when treatment has been interrupted and subsequently resumed.

Alongside dose flexibility, nutritional counseling was also considered an important and widely accepted component of tolerability management (42, 43, 45). Patients should be advised to avoid large meals, limit fluid intake during meals, and identify foods that exacerbate gastrointestinal symptoms. It is known that in some individuals, protein-rich foods may transiently intensify nausea, and this awareness may help optimize individualized dietary guidance. The main patient-directed suggestions for the management of gastrointestinal adverse events associated with GLP-1RA therapy are summarized in **Supplementary Table S2** in the **Supplementary Materials**.

Beyond tolerability considerations, the panel also highlighted the favorable qualitative pattern of weight loss associated with GLP-1RA therapy. Drawing on their clinical experience and supported by preliminary case-series observations (50), the panel noted that weight reduction induced by these agents appears to predominantly reflect a decrease in FM – including visceral adipose tissue – while SMM tends to be relatively preserved. Although some reduction in SMM may still occur, the overall effect tends to improve the “SMM-to-FM” ratio. Data from the STEP UP study indicate that muscle function is preserved, with weight loss primarily attributed to a reduction in FM (approximately 84%) (51). These findings are supported by evidence from the SEMALEAN study, which confirms that drug therapy combined with proper dietary management helps reduce FM while preserving SMM and improving muscle function (52). This observation reinforces the importance of combining pharmacotherapy with appropriate nutritional guidance and resistance-based physical activity to further support the preservation of SMM and optimize body composition outcomes (see the Appendix 1 “How to Prevent and Manage Cases of Sarcopenia” in **Supplementary Material**).

In this context, the panel strongly emphasized the importance of attention to nutritional status and bone health in patients receiving long-term pharmacological treatment for obesity. In particular, assessment of nutritional adequacy, including vitamin D status and adequate protein intake, should be considered according to individual clinical needs and risk factors. Bone health evaluations - such as Bone Mineral Density (BMD) by DEXA, with trabecular bone score (TBS) where clinically appropriate - may be considered in patients with risk factors for impaired bone health (53). Similarly, assessment of calcium–phosphorus metabolism (including calcium, phosphorus, parathyroid hormone, and, where appropriate, bone turnover markers such as C-terminal telopeptide of type 1 collagen - *CTX*) should be guided by the individual clinical context and bone-health risk profile. These measures, when clinically indicated, aim to mitigate the risk of impaired bone quality in patients at increased risk and to support the preservation of both skeletal and muscle health through adequate nutritional strategies, including sufficient protein intake and correction of vitamin D deficiency when present (54).

In addition, the panel stressed the importance of product quality and appropriate sourcing of GLP-1RA therapies as part of long-term safety considerations. Patients should be cautioned against purchasing medications through unauthorized online channels, given the well-documented risk of counterfeit or substandard products (“fake drugs”). The field of obesity management has historically been particularly vulnerable to such practices, and the growing demand for GLP-1RAs may further increase this risk. Moreover, the progressive expiration of patents for key compounds, particularly in regions with high biosimilar production such as China and India, may contribute to the expansion of unregulated markets. In this context, ensuring that patients access therapies through authorized and regulated supply chains represents a critical component of good clinical practice and long-term treatment safety.

Overall, the collective experience of the advisory board indicates that permanent treatment discontinuation due to adverse events is uncommon when a patient-centered and flexible management approach is adopted. With appropriate patient education, individualized dose modulation, and regular follow-up, GLP-1RA therapy can be safely and well tolerated over extended periods, reinforcing its role as a cornerstone of long-term obesity management.

Practical suggestions for long-term safety and tolerability of GLP-1RA therapy are summarized in **Table 1**.

## Conclusions and future directions

The expanding use of GLP-1RAs has transformed the therapeutic landscape for obesity management. The advisory board reaffirms that obesity should be approached as a chronic, relapsing disease necessitating long-term commitment rather than short-term interventions. Within this framework, authors believe that therapeutic success should move beyond a purely weight-centered paradigm and adopt a treat-to-benefit approach, in which treatment targets are defined according to the patient’s clinical phenotype and individual health motivations for seeking care. In the board’s opinion, key pillars for sustaining long-term benefits include:

- Comprehensive baseline phenotyping to guide clinical expectations.
- Structured maintenance phases and flexible dose titration to optimize durability.
- Continuous lifestyle integration to support metabolic health.

At the same time, real-world barriers – including tolerability issues, lifestyle constraints, and economic sustainability – should be proactively addressed to maintain long-term treatment adherence and prevent weight cycling (**Figure 1**, **Supplementary Figure S1**).

**Figure 1:**
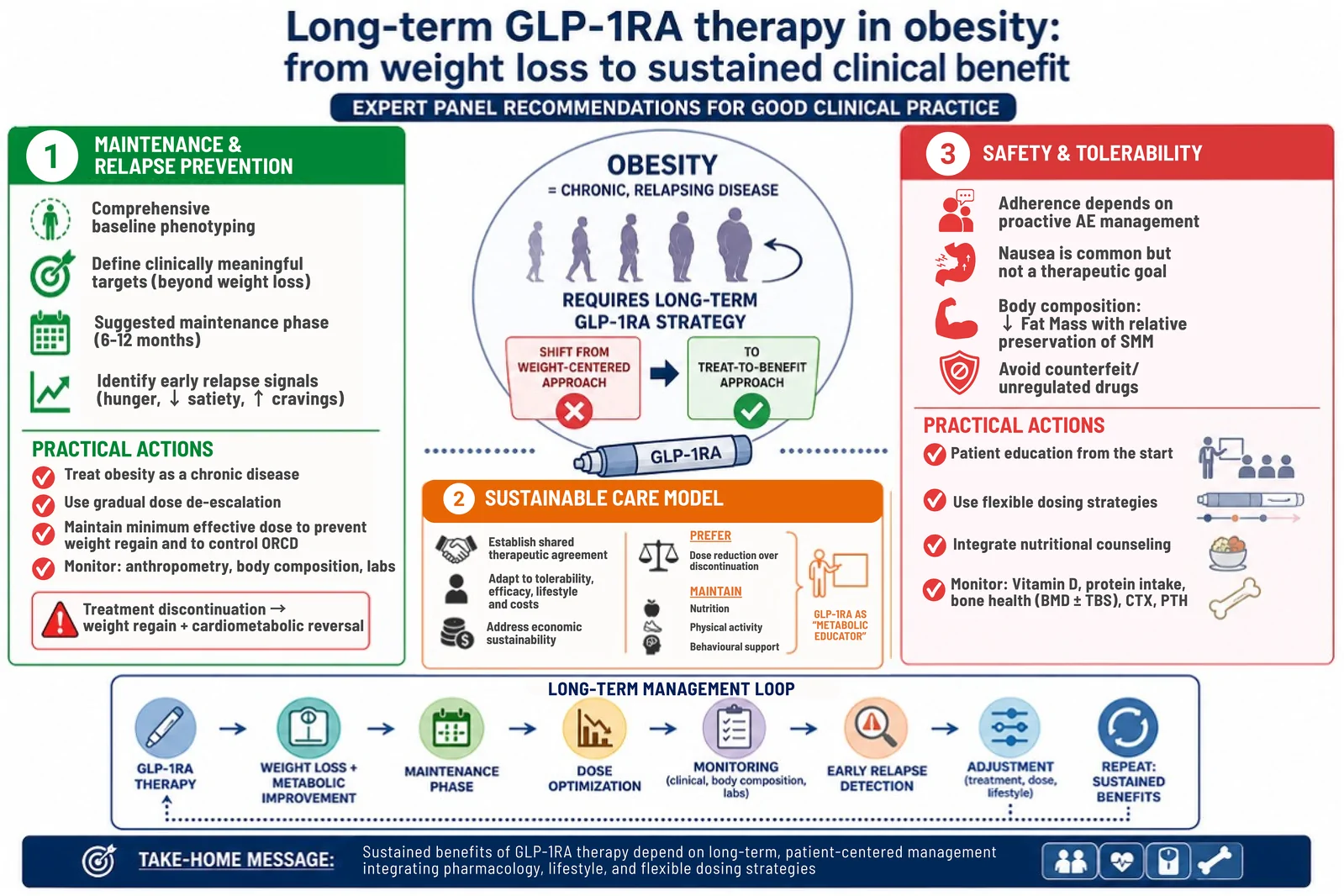
Summary of main recommendations from the expert panel for the long-term management of obesity with GLP-1 receptor agonists. The management process focuses on maintaining clinical benefits, preventing relapses, and providing sustained, patient-centered care.

Several areas warrant further investigation. This manuscript should be interpreted as an expert opinion rather than a formal consensus statement. Accordingly, the proposed recommendations reflect collective clinical experience and are intended to support, rather than replace, individualized clinical judgment and locally approved prescribing information. The advisory board proposes collaborative systematic analysis of real-world data from clinical databases to validate the practical recommendations proposed in this paper and generating additional evidence on long-term treatment patterns, maintenance strategies, dose adjustments, and clinical outcomes in routine care. Such analyses will facilitate the identification of predictors of treatment durability, allowing for more precise identification of patient subgroups most likely to derive long-term benefit from specific management approaches.

## Data Availability

The original contributions presented in the manuscript are included in the article/**Supplementary Material**. Further inquiries can be directed to the corresponding author/s.

## References

[1] Bifulco M Pagano C Di Zazzo E . Obesity legally recognised as a chronic disease in Italy. Lancet Diabetes Endocrinol. (2025) 13:1001–2. doi: 10.1016/S2213-8587(25)00326-2 41138743

[2] Kivimäki M Kuosma E Ferrie JE Luukkonen R Nyberg ST Alfredsson L . Overweight, obesity, and risk of cardiometabolic multimorbidity: pooled analysis of individual-level data for 120 813 adults from 16 cohort studies from the USA and Europe. Lancet Public Health. (2017) 2:e277–85. doi: 10.1016/S2468-2667(17)30074-9 28626830 PMC5463032

[3] Afshin A Forouzanfar MH Reitsma MB Sur P Estep K GBD 2015 Obesity Collaborators . Health effects of overweight and obesity in 195 countries over 25 years. N Engl J Med. (2017) 377:13–27. doi: 10.1056/NEJMoa1614362 28604169 PMC5477817

[4] Nauck MA Tuttle KR Tschöp MH Blüher M . GLP-1 receptor agonists and next-generation incretin-based medications: metabolic, cardiovascular, and renal benefits. Lancet. (2026) 407:892–908. doi: 10.1016/S0140-6736(25)02105-1 41547366

[5] Lincoff AM Brown-Frandsen K Colhoun HM Deanfield J Emerson SS Esbjerg S . Semaglutide and cardiovascular outcomes in obesity without diabetes. N Engl J Med. (2023) 389:2221–32. doi: 10.1056/NEJMoa2307563 37952131

[6] Moiz A Filion KB Tsoukas MA Yu OHY Peters TM Eisenberg MJ . The expanding role of GLP-1 receptor agonists: a narrative review of current evidence and future directions. eClinicalMedicine. (2025) 86:103363. doi: 10.1016/j.eclinm.2025.103363 40727007 PMC12303005

[7] Scragg J Koutoukidis DA Dirksen C Heitmann BL Jebb SA . The societal implications of using glucagon-like peptide-1 receptor agonists for the treatment of obesity. Med. (2025) 6:100805. doi: 10.1016/j.medj.2025.100805 40834863

[8] Salerno ENM Fumarulo I Garramone B Vaccarella M Ierardi C Burzotta F . GLP1-RAs: long-term use versus discontinuation events of an emerging therapy for obesity and cardiovascular diseases. Curr Probl Cardiol. (2026) 51:103213. doi: 10.1016/j.cpcardiol.2025.103213 41297647

[9] Guo H Yang J Huang J Xu L Lv Y Wang Y . Comparative efficacy and safety of GLP-1 receptor agonists for weight reduction: A model-based meta-analysis of placebo-controlled trials. Obes Pillars. (2025) 13:100162. doi: 10.1016/j.obpill.2025.100162 39980735 PMC11840199

[10] Moiz A Filion KB Toutounchi H Tsoukas MA Yu OHY Peters TM . Efficacy and safety of glucagon-like peptide-1 receptor agonists for weight loss among adults without diabetes: A systematic review of randomized controlled trials. Ann Intern Med. (2025) 178:199–217. doi: 10.7326/ANNALS-24-01590 39761578

[11] Thomsen RW Mailhac A Løhde JB Pottegård A . Real-world evidence on the utilization, clinical and comparative effectiveness, and adverse effects of newer GLP-1RA-based weight-loss therapies. Diabetes Obes Metab. (2025) 27:66–88. doi: 10.1111/dom.16364 40196933 PMC12000858

[12] Tzang C-C Wu P-H Luo C-A Chen Z-T Lee Y-T Huang ES . Metabolic rebound after GLP-1 receptor agonist discontinuation: a systematic review and meta-analysis. eClinicalMedicine. (2025) 90:103680. doi: 10.1016/j.eclinm.2025.103680 41399474 PMC12702299

[13] Budini B Luo S Tam M Stead I Lee A Akrami A . Trajectory of weight regain after cessation of GLP-1 receptor agonists: a systematic review and nonlinear meta-regression. eClinicalMedicine. (2026) 93:103796. doi: 10.1016/j.eclinm.2026.103796 41938838 PMC13043475

[14] Dhippayom T Meraz M Lee H Hur C Inadomi JM Veettil SK . GLP-1 receptor agonists for treating obesity without diabetes: A systematic review and meta-analysis of economic evaluations. Diabetes Obes Metab. (2026) 28:1339–49. doi: 10.1111/dom.70322 41365841 PMC12803668

[15] Berg S Stickle H Rose SJ Nemec EC . Discontinuing glucagon-like peptide-1 receptor agonists and body habitus: a systematic review and meta-analysis. Obes Rev. (2025) 26:e13929. doi: 10.1111/obr.13929 40186344

[16] Manne-Goehler J Teufel F Venter WDF . GLP-1 receptor agonists and the path to sustainable obesity care. JAMA Intern Med. (2025) 185:8. doi: 10.1001/jamainternmed.2024.3579 39401020

[17] Sharma AM Kushner RF . A proposed clinical staging system for obesity. Int J Obes. (2009) 33:289–95. doi: 10.1038/ijo.2009.2 19188927

[18] Catapano A Trinchese G Cimmino F Petrella L D’Angelo M Di Maio G . Impedance analysis to evaluate nutritional status in physiological and pathological conditions. Nutrients. (2023) 15:2264. doi: 10.3390/nu15102264 37242147 PMC10224243

[19] Blüher M . An overview of obesity-related complications: The epidemiological evidence linking body weight and other markers of obesity to adverse health outcomes. Diabetes Obes Metab. (2025) 27:3–19. doi: 10.1111/dom.16263 40069923 PMC12000860

[20] McGowan B Ciudin A Baker JL Busetto L Dicker D Frühbeck G . Framework for the pharmacological treatment of obesity and its complications from the European Association for the Study of Obesity (EASO). Nat Med. (2025) 31:3229–32. doi: 10.1038/s41591-025-03765-w 41039115

[21] West S Scragg J Aveyard P Oke JL Willis L Haffner SJP . Weight regain after cessation of medication for weight management: systematic review and meta-analysis. BMJ. (2026) 392:e085304. doi: 10.1136/bmj-2025-085304 41500720 PMC12776922

[22] World Health Organization . ICD-11 for Mortality and Morbidity Statistics: 5B81 Obesity (2024). Available online at: https://icd.who.int/browse/2024-01/mms/en#149403041 (Accessed March 10, 2026).

[23] World Health Organization . WHO Guideline on the Use of Glucagon-Like Peptide-1 (GLP-1) Therapies for the Treatment of Obesity in Adults (2025). Available online at: https://app.magicapp.org/#/guideline/LrRxrL.

[24] Celletti F Farrar J De Regil L . World Health Organization guideline on the use and indications of glucagon-like peptide-1 therapies for the treatment of obesity in adults. JAMA. (2026) 335:434. doi: 10.1001/jama.2025.24288 41324410

[25] Natali LD De Oliveira LGMS Barbosa ICDQ Portilho NDP . Cardiometabolic effects of GLP-1 analogs in obese and overweight patients with preexisting cardiovascular disease: A systematic review and meta analysis of randomized controlled trials. Heart Lung. (2025) 74:174–9. doi: 10.1016/j.hrtlng.2025.07.009 40682989

[26] Rubino D Abrahamsson N Davies M Hesse D Greenway FL Jensen C . Effect of continued weekly subcutaneous semaglutide vs placebo on weight loss maintenance in adults with overweight or obesity: The STEP 4 randomized clinical trial. JAMA. (2021) 325:1414–25. doi: 10.1001/jama.2021.3224 33755728 PMC7988425

[27] Wong M Wu A Garhe PK Biermann M . Reduced-frequency GLP1 therapy maintains weight, body composition, and metabolic syndrome improvements: a case series. Obesity. (2026) 34(Suppl 1):112–20. doi: 10.1002/oby.70137 41732031 PMC13250739

[28] Pampanelli S Terzoni S Pantanetti P Di Loreto C Mazzieri A Cangelosi G . Flexible dose of semaglutide reduces early discontinuation while maintaining comparable outcomes in obese patients: FLEX - SEMA 2.4 mg, an Italian real-world study. Diabetes Obes Metab. (2026) 28:3623–31. doi: 10.1111/dom.70538 41725422 PMC13071210

[29] Wegovy - Prescribing Information. Available online at: https://www.wegovy.com/prescribing-information.html (Accessed March 10, 2026).

[30] Gudzune KA Apovian CM Aroda VR Aronne LJ Balapattabi K American Diabetes Association Professional Practice Committee for Obesity* . Pharmacologic treatment of obesity in adults: Standards of care in overweight and obesity. BMJ Open Diabetes Res Care Care. (2026) 1:5–36. doi: 10.2337/doci25-0008 PMC12815056 41529914

[31] Sievenpiper JL Ard J Blüher M Chen W Dixon JB Fitch A . Nutritional and lifestyle supportive care recommendations for management of obesity with GLP-1 - based therapies: An expert consensus statement using a modified Delphi approach. Obes Pillars. (2026) 17:100228. doi: 10.1016/j.obpill.2025.100228 41502845 PMC12768930

[32] Codella R Senesi P Luzi L . GLP-1 agonists and exercise: the future of lifestyle prioritization. Front Clin Diabetes Healthc. (2025) 6:1720794. doi: 10.3389/fcdhc.2025.1720794 41367404 PMC12683586

[33] Chu J Zhang H Wu Y Huang Y Zhu T Zhou Z . Efficacy of lifestyle modification combined with GLP-1 receptor agonists on body weight and cardiometabolic biomarkers in individuals with overweight or obesity: a systematic review and meta-analysis. eClinicalMedicine. (2025) 88:103464. doi: 10.1016/j.eclinm.2025.103464 40926900 PMC12414836

[34] Abdelrahman RM Musa TH Arbab IA Suliman MH Ahmed EO Mohamed AN . Harnessing GLP-1 receptor agonists for obesity treatment: Prospects and obstacles on the horizon. J Obes. (2025) 2025:9919810. doi: 10.1155/jobe/9919810 41333115 PMC12668848

[35] Cengiz A Wu CC Lawley SD . Alternative dosing regimens of GLP-1 receptor agonists may reduce costs and maintain weight loss efficacy. Diabetes Obes Metab. (2025) 27:2251–8. doi: 10.1111/dom.16229 39950222 PMC11885104

[36] Knop FK Aroda VR Do Vale RD Holst-Hansen T Laursen PN Rosenstock J . Oral semaglutide 50 mg taken once per day in adults with overweight or obesity (OASIS 1): a randomised, double-blind, placebo-controlled, phase 3 trial. Lancet. (2023) 402:705–19. doi: 10.1016/S0140-6736(23)01185-6 37385278

[37] Wharton S Lingvay I Bogdanski P Duque Do Vale R Jacob S Karlsson T . Oral semaglutide at a dose of 25 mg in adults with overweight or obesity. N Engl J Med. (2025) 393:1077–87. doi: 10.1056/NEJMoa2500969 40934115

[38] Kadowaki T Heftdal LD Ko H-J Overvad M Shimomura I Thamattoor UK . Oral semaglutide in an East Asian population with overweight or obesity, with or without type 2 diabetes: The OASIS 2 randomized clinical trial. JAMA Intern Med. (2025) 185:1206. doi: 10.1001/jamainternmed.2025.3599 40758358 PMC12322823

[39] Kunutsor SK Seidu S . Safety and tolerability of glucagon-like peptide-1 receptor agonists: a state-of-the-art narrative review. Drugs. (2026) 86:11–36. doi: 10.1007/s40265-025-02263-0 41351656

[40] Chao AM Gilden A Wadden TA . Glucagon-like peptide-1 receptor agonists for obesity: Growing popularity met with growing questions over safety. PloS Med. (2026) 23:e1004871. doi: 10.1371/journal.pmed.1004871 41533690 PMC12803457

[41] Stone D Playdon M Hursting SD Tan AC . Evaluating the evolving real-world adverse events of GLP-1RAs using FDA Adverse Event Reporting System (FAERS). Obes (Silver Spring). (2026) 34(Suppl 2):101–8. doi: 10.1002/oby.70176 41797511 PMC13535732

[42] Gorgojo-Martínez JJ Mezquita-Raya P Carretero-Gómez J Castro A Cebrián-Cuenca A De Torres-Sánchez A . Clinical recommendations to manage gastrointestinal adverse events in patients treated with GLP-1 receptor agonists: a multidisciplinary expert consensus. JCM. (2022) 12:145. doi: 10.3390/jcm12010145 36614945 PMC9821052

[43] Alhazmi A Le Roux CW . Do no harm: managing nausea and vomiting in GLP-1 based obesity therapies. Front Endocrinol. (2026) 17:1788698. doi: 10.3389/fendo.2026.1788698 41852477 PMC12992036

[44] Yılmaz N Bastemir M . Gastrointestinal adverse effects of GLP-1 and dual GLP-1/GIP receptor agonists: a comprehensive update in diabetic and obese populations. Diabetes Metab Syndr Obes. (2026) 19:584175. doi: 10.2147/DMSO.S584175 41884101 PMC13012139

[45] Wharton S Davies M Dicker D Lingvay I Mosenzon O Rubino DM . Managing the gastrointestinal side effects of GLP-1 receptor agonists in obesity: recommendations for clinical practice. Postgrad Med. (2022) 134:14–9. doi: 10.1080/00325481.2021.2002616 34775881

[46] Eldor R Avraham N Rosenberg O Shpigelman M Golan-Cohen A Cukierman-Yaffe T . Gradual titration of semaglutide results in better treatment adherence and fewer adverse events: a randomized controlled open-label pilot study examining a 16-week flexible titration regimen versus label-recommended 8-week semaglutide titration regimen. Diabetes Care. (2025) 48:1607–11. doi: 10.2337/dc25-0690 40673973

[47] Kim JA Yoo HJ . Exploring the side effects of GLP-1 receptor agonist: to ensure its optimal positioning. Diabetes Metab J. (2025) 49:525–41. doi: 10.4093/dmj.2025.0242 40631457 PMC12270588

[48] Wilding JPH Batterham RL Calanna S Davies M Van Gaal LF Lingvay I . Once-weekly semaglutide in adults with overweight or obesity. N Engl J Med. (2021) 384:989–1002. doi: 10.1056/NEJMoa2032183 33567185

[49] Wharton S Calanna S Davies M Dicker D Goldman B Lingvay I . Gastrointestinal tolerability of once-weekly semaglutide 2.4 mg in adults with overweight or obesity, and the relationship between gastrointestinal adverse events and weight loss. Diabetes Obes Metab. (2022) 24:94–105. doi: 10.1111/dom.14551 34514682 PMC9293236

[50] Tinsley GM Nadolsky S . Preservation of lean soft tissue during weight loss induced by GLP-1 and GLP-1/GIP receptor agonists: a case series. SAGE Open Med Case Rep. (2025) 13:2050313X251388724. doi: 10.1177/2050313X251388724 41122508 PMC12536186

[51] Alissou M Demangeat T Folope V Van Elslande H Lelandais H Blanchemaison J . Impact of semaglutide on fat mass, lean mass and muscle function in patients with obesity: The SEMALEAN study. Diabetes Obes Metab. (2026) 28:112–21. doi: 10.1111/dom.70141 41068996 PMC12673431

[52] Wharton S Freitas P Hjelmesæth J Kabisch M Kandler K Lingvay I . Once-weekly semaglutide 7·2 mg in adults with obesity (STEP UP): a randomised, controlled, phase 3b trial. Lancet Diabetes Endocrinol. (2025) 13:949–63. doi: 10.1016/S2213-8587(25)00226-8 40961952

[53] Urbina J Salinas‐Ruiz LE Valenciano C Clapp B . Micronutrient and nutritional deficiencies associated with GLP ‐1 receptor agonist therapy: A narrative review. Clin Obes. (2026) 16:e70070. doi: 10.1111/cob.70070 41549912

[54] Mozaffarian D Agarwal M Aggarwal M Alexander L Apovian CM Bindlish S . Nutritional priorities to support GLP ‐1 therapy for obesity: A joint advisory from the American College of Lifestyle Medicine, the American Society for Nutrition, the Obesity Medicine Association, and The Obesity Society. Obesity. (2025) 33:1475–503. doi: 10.1002/oby.24336 40445127 PMC12304835

