# Supplementary material for: Long-term GLP-1 receptor agonist therapy in obesity: expert panel recommendations for good clinical practice
Source: Front Endocrinol (Lausanne). 2026 Sep 28;17:1907270. doi: 10.3389/fendo.2026.1907270 (PMC13654747; doi:10.3389/fendo.2026.1907270)
Supplement: Supplementary file 1 [file SupplementaryFile1.docx]

**Supplementary Material**

**Table S1. Practical tips for patient follow-up and adherence to treatment**

|  | Practical tips for patient follow-up and adherence to treatment |
| --- | --- |
| Baseline assessment | - Anthropometric parameters: weight, height, BMI, WC, WHtR - Body composition by BIA (or DXA): FFM, SMM, FM, TBW, ECW, ICW, PhA - Presence of ORCD - Blood tests: Blood glucose, insulinemia, glycated haemoglobin, lipid profile, uric acid, hs-CRP, complete blood count, TSH reflex, vitamin B12, gamma-GT, ALT, AST - Nutritional assessment and dietary advice - Explanation of the diagnostic and treatment process and any potential side effects - Abdominal ultrasound: to check for the presence of liver steatosis and assess the gallbladder |
| After 2 and 4 months, consider  Telemedicine/email | - Adherence to treatment and drug dosage - Adherence to diet and physical activity - Body weight in kg, % weight loss - Any side effect |
| After 6, 9 and 12 months | - Adherence to treatment and drug dosage - Adherence to diet and physical activity - Anthropometric indices: Weight, Height, BMI, WC, WHtR, % weight loss - Body composition by BIA (or DEXA where applicable), FFM, SMM, FM, TBW, ECW, ICW, PhA - Blood tests: Blood glucose, insulinemia, Glycated haemoglobin, lipid profile, uric acid, hs-CRP, complete blood count, TSH reflex (where applicable), vitamin B12, Gamma-GT, ALT, AST - Nutritional assessment and dietary advice - re-avaluate and possibly reduce all relevant pharmacological treatment (antihypertensive drugs, thyroxine treatment etc) - abdominal ultrasound - Any side effect |
| Baseline assessment and after 12 months | **In older people (aged 65 and over) with osteoporosis or who have risk factors, with weight loss > than 10%**   - Family history of osteoporosis and/or fractures - BMD (and TBS) by DXA - Morphometry / X-ray of the thoracic and lumbar spine - Bone turnover markers: calcium, albumin, phosphorus, PTH, 25(OH)D, CTX, (P1NP), alkaline phosphatase |
| *Abbreviations*: 25(OH)D (25-hydroxyvitamin D); ALT = Alanine Aminotransferase; AST = Aspartate Aminotransferase; BIA (Bioelectrical Impedance Analysis); BMD (Bone Mineral Density); BMI (Body Mass Index); CTX (C-terminal telopeptide of type 1 collagen); DEXA (Dual-energy X-ray Absorptiometry); ECW (Extracellular Water); FFM (Fat-Free Mass); FM (Fat Mass); hs-CRP (high sensitivity-C reactive protein); ICW (Intracellular Water); MASLD (Metabolic Dysfunction-associated Steatotic Liver Disease); ORCD (Obesity-Related Complications and Diseases); P1NP (Procollagen type 1 N-propeptide); PhA (Phase Angle); PTH (Parathyroid hormone); SMM (Skeletal Muscle Mass); TBS (Trabecular Bone Score); TBW (Total Body Water); TSH (Thyroid Stimulating Hormone); WC (Waist Circumference); WHtR (Waist-to-Height Ratio) | |

**Table S2. Dietary and behavioral recommendations for managing GLP1 RA- induced gastrointestinal side effects**

|  | Dietary and Behavioral Recommendations |
| --- | --- |
| Meal Patterns & Portions | - Consume small, frequent meals throughout the day - Reduce overall portion sizes to prevent gastric distension - Eat slowly and mindfully, stopping immediately upon reaching satiety - Eat only when experiencing genuine physiological hunger |
| Dietary Choices | - Avoid high-fat, fried, spicy, or heavily seasoned foods - Prefer easily digestible food - Prefer simple cooking methods such as steaming, baking, boiling, or grilling - Limit intake of alcohol and carbonated beverages - Prioritize water and sugar-free fluids for hydration - In case of nausea, consume ginger or mint to mitigate symptoms and prioritize bland foods - In case of diarrhea, avoid extreme food temperatures and eliminate products containing polyols (e.g., sorbitol, mannitol, xylitol), such as sugar-free candies and gum, to avoid osmotic worsening of symptoms |
| Fluid Management | - Maintain adequate hydration with small sips throughout the day - Increase fluid intake 30–60 minutes before or after meals, rather than during meals - Avoid using a straw to drink |
| Behavioral habits | - Avoid distractions while eating - Avoid lying down or engaging in intense physical activity immediately after eating - Take a short walk in fresh air after meals - Maintain regular physical activity to stimulate intestinal motility |
| Clinical Compliance | - Strictly follow the prescribed dose-escalation schedule to minimize gastrointestinal intolerance - Maintain a food diary to identify and monitor individual triggers |

**Appendix 1: How to Prevent and Manage Cases of Sarcopenia**

The increasing use of anti-obesity medications has raised many questions and challenges in the practical management of patients. In particular, the management of the loss of muscle mass during therapy with GLP-1 analogues is one of the “hottest” topic nowadays^1^. After all, the goal of weight loss is not only a reduction in absolute body weight, but also the optimization of body composition and tissue function.

When weight is lost, in any weight-loss pathway (even purely dietary), there is inevitably a reduction in both lean mass, skeletal muscle mass and fat mass. Therefore, it is important to implement all strategies to identify individuals at higher risk and to prevent/correct any reduction in muscle mass in terms of muscle strength, muscle “quantity,” and performance. In other words, the risk of sarcopenia must be reduced. This risk is particularly relevant in certain groups, such as individuals over the age of 50, those with obesity or type 2 diabetes, and individuals with a sedentary lifestyle or who are already frail.

To identify sarcopenia, it is possible to refer to the diagnostic criteria established by the European Working Group on Sarcopenia in Older People^2^, adapted to patients undergoing pharmacological treatment for obesity: reduced muscle strength, decreased muscle mass, and impaired physical performance. Tools such as dynamometry for strength, Dual-energy X-ray absorptiometry (DXA) or Bioelectrical Impedance Analysis (BIA) for body composition, and functional tests such as gait speed or handgrip strength can be used for this purpose. It is important to note the respective roles and limitations of BIA and DXA in the monitoring of muscle mass. Indeed, while DXA provides a more standardized assessment of total and appendicular lean mass and may therefore be preferable when a more accurate evaluation of body composition is required or when the diagnosis of sarcopenia is uncertain, BIA is more accessible, inexpensive, rapid, and suitable for repeated assessments in routine clinical practice. Nevertheless, BIA estimates muscle mass indirectly through predictive equations and its results may be influenced by hydration status, recent food and fluid intake, physical activity, edema, device characteristics, and the reference equation used. Accordingly, BIA-derived values should not be considered interchangeable with DXA measurements. Longitudinal monitoring should preferably be performed using the same technique, device, and standardized measurement conditions. Changes in BIA-derived muscle mass should be interpreted together with muscle strength, physical performance, nutritional status, and the overall clinical context, rather than being used as an isolated indicator of sarcopenia. ^3,4^

Once the risk or presence of sarcopenia has been identified, it becomes crucial to adopt protective strategies. Interventions should begin as soon as clinically relevant risk factors, declining muscle function, or diagnostic features of sarcopenia are identified. Among these, physical exercise plays a central role, particularly resistance training, which is the most effective in preserving and increasing muscle mass. Its implementation should be individualized according to age, baseline physical function, previous exercise experience, comorbidities, musculoskeletal limitations, and the presence or risk of sarcopenia^5^.

Resistance training should be regarded as the primary exercise strategy for preserving lean mass and muscle function during pharmacological weight loss, as aerobic activity alone, although beneficial for cardiorespiratory fitness, glycaemic control, and adiposity reduction, does not provide an equivalent anabolic stimulus. In clinical practice, however, implementation must account for the low adherence commonly observed in patients with severe or sarcopenic obesity. For this reason, recommendations should emphasize accessible and adaptable approaches, including chair-based or aquatic resistance exercise, home-based programmes using resistance bands or body-weight exercises, behavioral support, and telehealth or supervised follow-up when available. In general, whole-body resistance training involving the major muscle groups should be performed two to three times per week, with gradual progression in resistance and sufficient effort to stimulate adaptation. The specific format should be individualized according to age, baseline physical capacity, joint pain, balance, confidence, access to equipment, and previous exercise experience. The key objective is not to prescribe a uniform programme, but to ensure that each patient receives a feasible, progressive, and sufficiently challenging resistance stimulus to help preserve muscle mass, strength, and functional reserve during ongoing weight reduction. ^5,6^

The program should be adjusted if the patient experiences persistent pain, marked fatigue, functional decline, dizziness, chest symptoms, or other clinically relevant limitations. Resistance training should be combined with adequate protein intake, nutritional monitoring, and, where appropriate, aerobic and balance exercises as part of a comprehensive strategy to preserve muscle and physical function.^5,6^

At the same time, adequate nutritional support is essential, with sufficient protein intake, generally ranging between 1.0 and 1.5 grams per kilogram of body weight per day, prioritizing proteins with high biological value and making them the main component of the meal (followed by carbohydrates, fats, and fiber)^7^.

Modulate weight loss not to exceed 1kg per week, particularly in patients at risk for sarcopenia.

| Step | Clinical Objective | Assessment Criteria and Recommended Interventions |
| --- | --- | --- |
| Step 1 | Identification of patients at risk | Patients should be considered at risk if they meet one or more of the following criteria:  - Age > 50 years  - Presence of obesity and/or type 2 diabetes mellitus (T2DM)  - Sedentary lifestyle and/or clinical signs of frailty |
| Step 2 | Diagnosis of sarcopenia according to EWGSOP2 criteria | Sarcopenia diagnosis should follow a stepwise approach:  **Assessment of muscle strength:**  - Handgrip strength < 27 kg in men or < 16 kg in women, and/or  - Chair stand test > 15 seconds (5 repetitions)  **If reduced strength is confirmed, assessment of muscle mass is required:**  - Dual-energy X-ray absorptiometry (DXA): < 7.0 kg/m² in men or < 5.5 kg/m² in women  The presence of both reduced muscle strength and low muscle mass confirms the diagnosis of sarcopenia. |
| Step 3 | Implementation of protective strategies | The following interventions are recommended as soon as clinically relevant risk factors, declining muscle function, or diagnostic features of sarcopenia are identified, with the aim of risk or progression of sarcopenia being mitigated:  - Combined resistance and aerobic exercise training  - Optimization of dietary protein intake  - Adjustment of meal timing (e.g., small, frequent meals)  - Periodic monitoring of body composition, when feasible  - Individualization of GLP-1 receptor agonist (GLP-1 RA) therapy in cases of rapid weight loss and/or muscle mass reduction |

Finally, we should not forget personalization: the obesity treatment medications are a highly effective tool for weight loss, but their use must be accompanied by an integrated approach that takes muscle health into account, in order to maximize benefits and reduce the risk of sarcopenia and/or other side effects.

**Appendix 2: How to Prevent and Manage Gallbladder Stones**

Before starting treatment with GLP-1RA assess the presence of gallbladder stones.

If patients show gallbladder stones, treatment can be started but the patient should be treated with ursodeoxycholic acid (300 b.i.d. at lunch at supper) and reevaluated after 6 months.

If a patient does not show gallbladder stones, reassess in 6 months.

To reduce the risk of gallbladder stones formation, modulate weight loss not to exceed 1kg per week.

If at six months patients show formation of gallbladder stones start treatment as above.

**Figure S2. Practical clinical decision pathway for the long-term management of GLP-1 receptor agonist therapy in patients with obesity.**

The pathway begins with multidimensional baseline phenotyping, including anthropometric and body composition assessment, obesity severity and complications, cardiometabolic risk, behavioral and psychological drivers of weight gain, patient expectations, and treatment sustainability. Individualized therapeutic goals should be defined according to a treat-to-benefit approach and periodically reassessed throughout treatment. Once clinically meaningful outcomes have been achieved, GLP-1RA therapy should generally be continued during a maintenance and consolidation phase. Dose de-escalation may subsequently be considered on an individualized basis in patients with sustained clinical stability, tolerability concerns, patient preference, or economic and access-related constraints. Dose reduction should be gradual, with continued monitoring of weight trajectory, body composition, hunger, satiety, obesity-related complications, tolerability, adherence, and quality of life. The re-emergence of hunger, loss of satiety, clinically meaningful weight regain, or worsening of metabolic or functional outcomes should prompt reassessment of adherence and other contributing factors and may warrant re-escalation to the previously effective and tolerated dose. Following a prolonged treatment interruption, cautious re-initiation and individualized re-titration may be appropriate, particularly in patients with previous gastrointestinal adverse events. The pathway is intended as expert-informed practical guidance and should be adapted to patient characteristics, available evidence, prescribing information, and local clinical practice.

**References**

1.	Ryan, D. H. New drugs for the treatment of obesity: do we need approaches to preserve muscle mass? *Rev. Endocr. Metab. Disord.* **26**, 805–813 (2025).

2.	Cruz-Jentoft, A. J. *et al.* Sarcopenia: revised European consensus on definition and diagnosis. *Age Ageing* **48**, 16–31 (2019).

3.	Guglielmi, G., Ponti, F., Agostini, M. *et al.* The role of DXA in sarcopenia. *Aging Clin Exp Res* **28**, 1047–1060 (2016). https://doi.org/10.1007/s40520-016-0589-3

4. Herda, A.A., Cleary, C.J. Agreement between multifrequency BIA and DXA for assessing segmental appendicular skeletal muscle mass in older adults. *Aging Clin Exp Res* **34**, 2789–2795 (2022).

5. Šantić, R. *et al*. Lean Mass and Musculoskeletal Preservation in GLP-1-Based Obesity Treatment: Nutrition, Exercise, Supplementation, and Monitoring Strategies. *Metabolites*.;**16**(6):364 (2026)

6. Sancho-Haro, E., *et al.* Optimizing Weight Loss in the GLP-1 Era: Preserving Muscle Mass, Function and Metabolic Health Through Precision Nutrition and Resistance Training. *Pharmaceuticals (Basel, Switzerland)*, ***19***(6), 897(2026)..

7. Ceasovschih, A. *et al.* Glucagon-like peptide-1 receptor agonists and muscle mass effects. *Pharmacol. Res.* **220**, 107927 (2025)
